# New synthesis of poly ortho-methoxyaniline nanostructures and its application to construct modified multi-wall carbon nanotube/graphite paste electrode for simultaneous determination of uric acid and folic acid

**DOI:** 10.1016/j.msec.2017.02.133

**Published:** 2017-06-01

**Authors:** Hossein Rajabi, Meissam Noroozifar

**Affiliations:** Analytical Research Laboratory, Department of Chemistry, University of Sistan and Baluchestan, Zahedan, P.O. Box 98155-674, Iran

**Keywords:** Polyortho-methoxyaniline nano structures, Electrocatalytic oxidation, Multi-wall carbon nanotubes, Uric acid, Folic acid

## Abstract

Uric acid (UA) and folic acid (FA) are compounds of biomedical interest. In humans, about 70% of daily uric acid disposal occurs via the kidneys, and in 5–25% of humans, impaired renal (kidney) excretion leads to hyperuricemia. Folate is another form folic acid of which is known as, is one of the B vitamins. It is used as a supplement by women to prevent neural tube defects developing during pregnancy. Polyortho-methoxyaniline nanostructures (POMANS) was synthesized with a new two phase (organic-water) synthesis method. The POMANS was characterized using transmission electron microscopy (TEM) and Fourier transform IR (FTIR). This polymer was used to construct a modified multi-wall carbon nanotube, graphite paste electrode (POMANS-MWCNT/GPE). Linear sweep voltammograms (LSV), cyclic voltammetry (CV) and chronoamperometry were used to investigate the suitability of polyortho-methoxyaniline with multi-wall carbon nanotubes paste electrode as a modifier for the electrocatalytic oxidation of UA and FA in aqueous solutions with various pHs. The results showed that POMANS-MWCNT/GPE had high anodic peak currents for the electrooxidation of UA and FA in pH 6.0.Under the optimized conditions, The catalytic peak currents obtained, was linearly dependent on the UA and FA concentrations in the range of 0.6–52 and 0.5–68 μM with two segments and the detection limits 0.157 and 0.113 μM for UA and FA were, respectively. Finally, the proposed method was also examined as a sensitive, simple and inexpensive electrochemical sensor for the simultaneous determination of UA and FA in real samples such as urine and serum.

## Highlights

• For the first time, POMANS was synthesized with a new method of two-phase organic & water.
• POMANS-MWCNT/GPE was used for simultaneous determination of UA and FA at optimum pH 6.0.
• Parameters n and α were also determined for UA and FA.
• Electrochemical simultaneous determination of UA and FA with modified electrode real samples.

## 1 Introduction

Conducting polymers (CPs) such as polyaniline, polypyrrole, polythiophene and their derivatives, has been used as modifier in electrochemical sensors, catalysts and electro chromic materials and many more, etc. [1], [2], [3], [4], [5]. Among different CPs, poly ortho-methoxyaniline (POMA)has been one of the most extensively studied polymers in fabrication of the sensor devices, because POMA has useful features including high electrical conductivity, good environmental stability, electrochemical redox activity even in neutral pH solutions [6], [7], [8], [9].To increase the surface area of catalysis, enhance catalytic activity and sensitivity due to the increase of surface, POMA has been supported by different material [10], [11].

Uric acid (2,6,8-trihydroxypurine) (UA) the end metabolic product of purine through the liver, is present in blood and urine. Abnormal UA level in a human body could be caused

by several diseases such as gout, hyperuricemia, Lesch–Nyan syndrome, as well as cardiovascular and chronic renal diseases. Hence, monitoring of the concentration of UA in biological fluids may be used as an early warning of the presence of these diseases. Electrochemical sensors for the determination of UA are simple, rapid, inexpensive and easy to use [12], [13], [14], [15], [16]. Folic acid, ((2*S*)-2-[(4-{[(2-amino-4-hydroxypteridin-6yl)methyl]amino}phenyl) formamido] pentanedioic acid), also known as vitamin B_c_, vitamin M and folacin, is a form of the water-soluble vitamin B_9_. FA deficiency causes failure to make the purines and thymine required for DNA synthesis. FA is necessary for cell development, for metabolism of specific biochemical reactions in the body and the metabolism of specific anticonvulsant drugs [17], [18], [19]. Since, UA and FA always co-exist in the human body fluids. So, it has essential for the simultaneous determination of UA and FA.

Carbon nanotubes (CNTs, includes multi-wall carbon nanotubes and single-wall carbon nanotubes), the new forms of elementary carbon, are composed of graphitic sheets rolled into closed concentric cylinders with diameter of nanometers and length of micrometers. Because of the special tube structure, CNTs possess several unique properties such as good electrical conductivity, high chemical stability, and extremely high mechanical strength [20]. The better performance of the CNTs electrode compared to other electrodes may be due to the carbon nanotube dimensions, the electronic structure, and the topologic defects present on the tube surface [21], [22]. Problems of low catalytic performance for analysis of toxic and biologic compounds with CNTs, nano composites and its hybrids are improved [23], [24]. It has been reported that carbon nanotube-modified electrodes were successfully applied to study and determine many biological compounds, drugs and toxic materials [25], [26], [27], [28].

To our knowledge, there are no similar studies on the polymerization of poly ortho-methoxyaniline nanostructures (POMANS) and its application as novel modifier in modified graphite paste electrode including MWCNTs for the simultaneous determination of uric acid and folic acid in aqueous solution. The present work describes the synthesis of POMA with a new two phase (organic-water) method. The electrochemistry behavior of POMANS-MWCNTs/GPE and investigation of its electrocatalytic effect on simultaneous determination of UA and FA was studied in details. It was found that the POMANS-MWCNTs/GPE showed an electrocatalytic activity towards the oxidation of UA and FA by enhancing its oxidation currents when compared to bare GPE and MWCNTs/GPE. Finally, in order to demonstrate the catalytic ability of the modified electrode in the determination of UA and FA in real samples, we examined this method for the voltammetric simultaneous determination of UA and FA in samples of serum and urine.

## 2 Experimental

### 2.1 Chemicals

Isobutyl methyl ketone (4-methyl-2-pentanone), potassium persulfate (K_2_S_2_O_8_), phosphoric acid, hydrochloric acid and sodium hydroxide with analytical grade were obtained from Merck company. Uric acid, folic acid, multi-wall carbon nanotubes, with nanotube diameters, OD = 20–30 nm, wall thickness = 1–2 nm, length = 0.5–2 μm and purity > 95%, graphite powder and high-viscosity paraffin oil were purchased from Aldrich. Ortho-methoxyaniline (OMA)was prepared from Aldrich and was purified immediately prior to use by passing small aliquots through an activated alumina column. All solutions were freshly prepared by doubly distilled water (DDW), purged with pure nitrogen gas (99.999%) before investigations.

### 2.2 Instrumentation

All electrochemical experiments were performed using a SAMA 500 Electroanalyser (SAMA Research Center, Iran)controlled by a personal computer. A platinum wire was used as the auxiliary electrode. A saturated calomel electrode (SCE) and POMANS-MWCNTs/GPE were used as the reference and working electrodes, respectively. A pH-meter (Metrohm 744 pH/ion meter) was used to read the pH of the buffered solutions. The FTIR spectra were measured in transmission mode using Valor III (JASCO) equipped with a MCT detector. TEM images were taken using a Philips CM120 transmission electron microscopy with 2.5 A° resolution.

### 2.3 Synthesis of poly ortho-methoxyaniline nano structures

In order to synthesis of poly ortho-methoxyaniline nano structures, we prepared two separate solutions in organic phase and aqueous phase; for organic phase, 1.5 mL of OMA (after the purified with activated alumina column) was added to 10mLisobutyl methyl ketone at acidic media (0.1 ml HCl as catalyzer) and the aqueous phase, 5.0 g of potassium persulfate in 50 mL of doubly distilled water was dissolved. Next, provided organic phase was added to aqueous phase and was placed in ultrasonic agitation for 2 h. The mixed solution was stirred by magnetic stirring apparatus at room temperature for 3 h. Then, by a decanter was separated aqueous phase from organic phase and dried at vacuum oven at 60 °C for 2 h for fling solvent, then prepared polymer was washed doubly distilled water and was dried at room temperature.

### 2.4 Preparation of the electrode

In the other to obtain the best conditions in the preparation of the POMANS-MWCNTs/GPE, we optimized the ratio of POMANS, MWCNTs and graphite powder. The POMANS-MWCNTs/GPE was prepared by hand-mixing of 0.80 g of graphite powder, 0.15 g POMANS and 0.05 g MWCNTs plus paraffin (~ 0.8 mL) and mixed well for 30 min until a uniformly wetted paste was obtained. This paste was then packed into the end of a glass tube. A copper wire inserted into the carbon paste provided an electrical contact. When necessary, a new surface was obtained by pushing an excess of paste out of the tube and polishing with weighing paper. For comparison, a modified CPE (MWCNTs/GPE) without POMANS and an unmodified GPE without both POMANS and MWCNTs were also prepared in the same method.

## 3 Results and discussion

### 3.1 Characterization of poly ortho-methoxyaniline nano structures

**Fig. 1:**
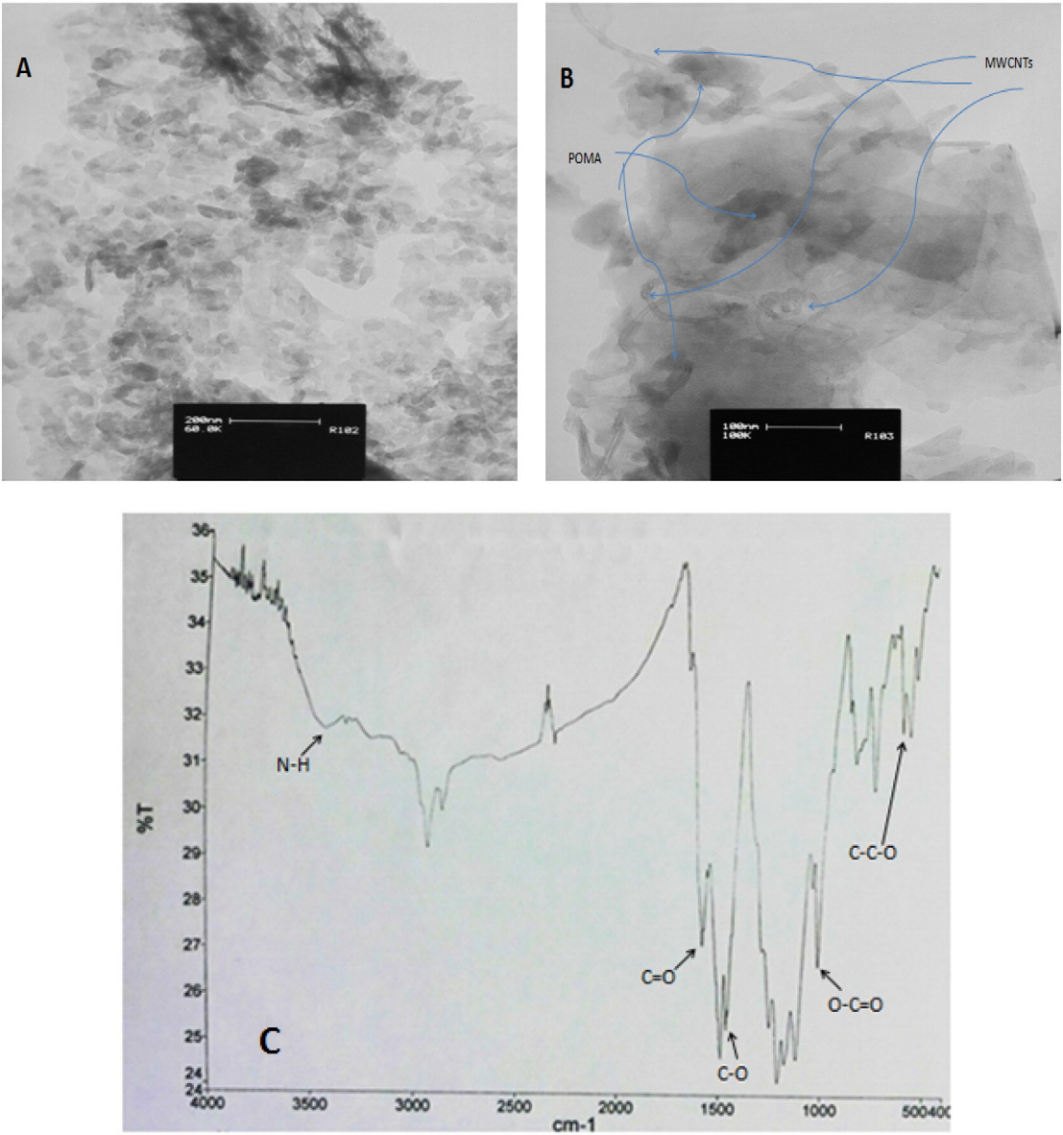
TEM images of (A) POMANS(B)POMANS-MWCNT/GPE, and (C) FTIR spectrum of POMANS.

The morphology of poly ortho-methoxyaniline nano structures was characterized by transmission electron microscopy and Fourier transform IR. As can be seen in Fig. 1A, the POMANS film shows homogeny morphology with the formation of aggregates. The aggregate size is in the range of several decades of nanometers. MWCNTs can be seen on the surface of GPE as is shown in Fig. 1B. Also, in addition to MWCNTs, nano structures from POMA at the surface of electrode are covered with graphite sheets mixed with aggregates of carbon particles (Fig. 1B). In this case a massive and packed layer has covered the electrode surface in a way that blocked the interlayer diffusion of the analyte species through the film. FTIR spectroscopy(Fig. 1C) evidences for presence of N—H, C=O, C—O, O—C=O and C—C—O at POMA sheet. The FTIR spectrum of POMA shows a N—H stretching vibrations at 3436.9 cm^− 1^, Presence of carbonyl group C=O at 1658.7 cm^− 1^, Corresponds to C—O group at 1433.0 & 1413.7 cm^− 1^, O—C=O in the plane deformation at 954.7 cm^− 1^, C—C—O in plane deformation at 613.3 cm^− 1^, stretching vibrations in quinoid-ring at 1658.7 cm^− 1^and Stretching vibrations of benzoid-ring at 1433.0 cm^− 1^ [29].

### 3.2 Electrochemical behavior of UA and FA on POMANS-MWCNT/GPE

**Fig. 2:**
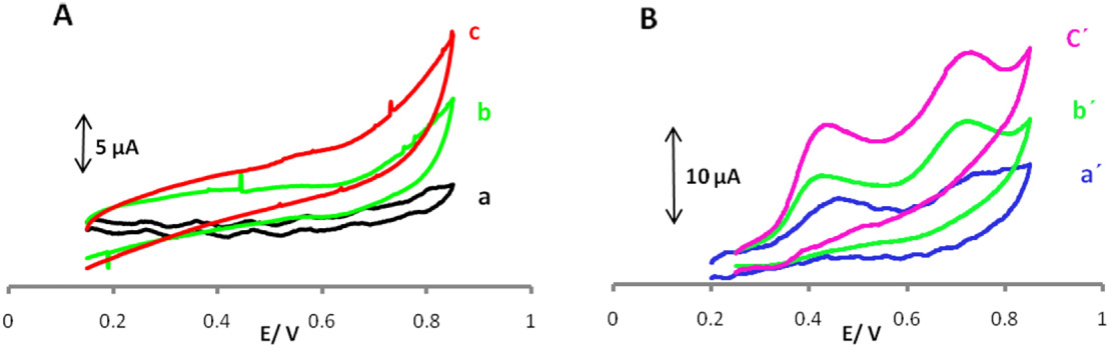
A. Cyclic voltammograms of (a) carbon paste electrode; (b) MWCNT/GPE and (c) POMANS-MWCNT/GPE in 0.1 M phosphate buffer (pH 6.0) at the scan rate of 50 mVs^− 1^ without UA and FA. B. Similar with of panel A in presence of 0.1 mM UA and 0.15 mM FA.

Cyclic voltammetry studies using three different electrodes, GPE, MWCNT/GPE, and POMANS-MWCNT/GPE, in buffered solution (blank) showed that in the entire potential range, no considerable peak can be seen (Fig. 2). Fig. 2B cyclic voltammograms were recorded for 0.1 mM UA and 0.15 mM FA at surface of noticed electrodes. As can be seen a small anodic peak currents for UA and FA at GPE, with added MWCNTs to GPE was seen (curve b′ in Fig. 2B) significantly increased the anodic peak currents compared with the GPE. However, it is obvious that the POMANS-MWCNT/GPE exhibits enhanced electrocatalytic oxidation with higher peak currents for the oxidation of UA and FA in comparison to the GPE and MWCNT/GPE (curve c′ in Fig. 2B)·The combination of POMANS and MWCNTs improved the electronic transport capacity and made the electron transfer easier between species and electrode.

### 3.3 Influence of solution pH

**Fig. 3:**
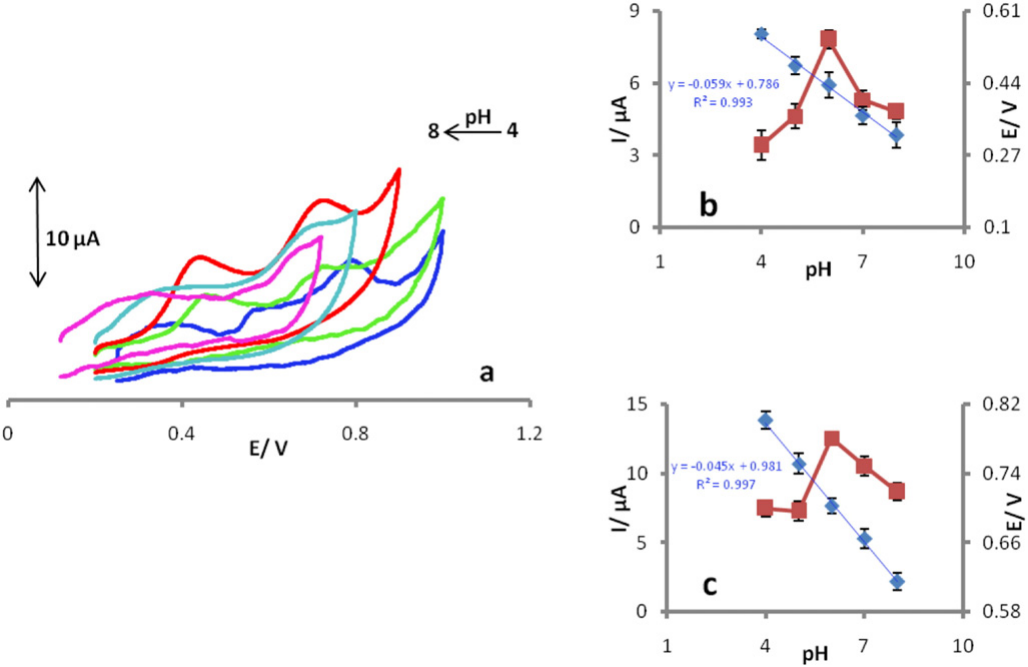
Influence of supporting electrolyte pH on peak current responses and peak potential responses of UA and FA at POMANS-MWCNT/GPE (a). Insertions show Current–pH curve &potential–pH curve for electrooxidation of UA (b) and FA (c). Cyclic voltammograms were measured scan rate 20 mV s^− 1^ with 25 μM UA and 50 μM FA.

The voltammetric investigations were performed in the pH range of 4.0–8.0 using 0.1 M phosphate buffer solution (PBS) as supporting electrolyte, containing 25 μM UA and 50 μM FA at scan rate 20 mV s^− 1^(Fig. 3a).As can be seen in Fig. 3b & c, the anodic peak potential shows a negative shift by increasing the solution pH with a slope value of 45.0 mV and 59.0 mV per pH unit for UA and FA, respectively (near to the theoretical slope, 59 mV per pH unit).From the results, it can be concluded that equal numbers of electrons and protons are involved in the electro-oxidation of UA and FA on the surface of the POMANS-MWCNT/GPE [13], [18]. The anodic peak currents of UA and FA reach their maximum values at pH 6.0 (Fig. 3b & c). Therefore, all voltammetric determinations have been performed in a phosphate buffer solution of pH 6.0, as the optimum pH.

### 3.4 Effect of scan rate

**Fig. 4:**
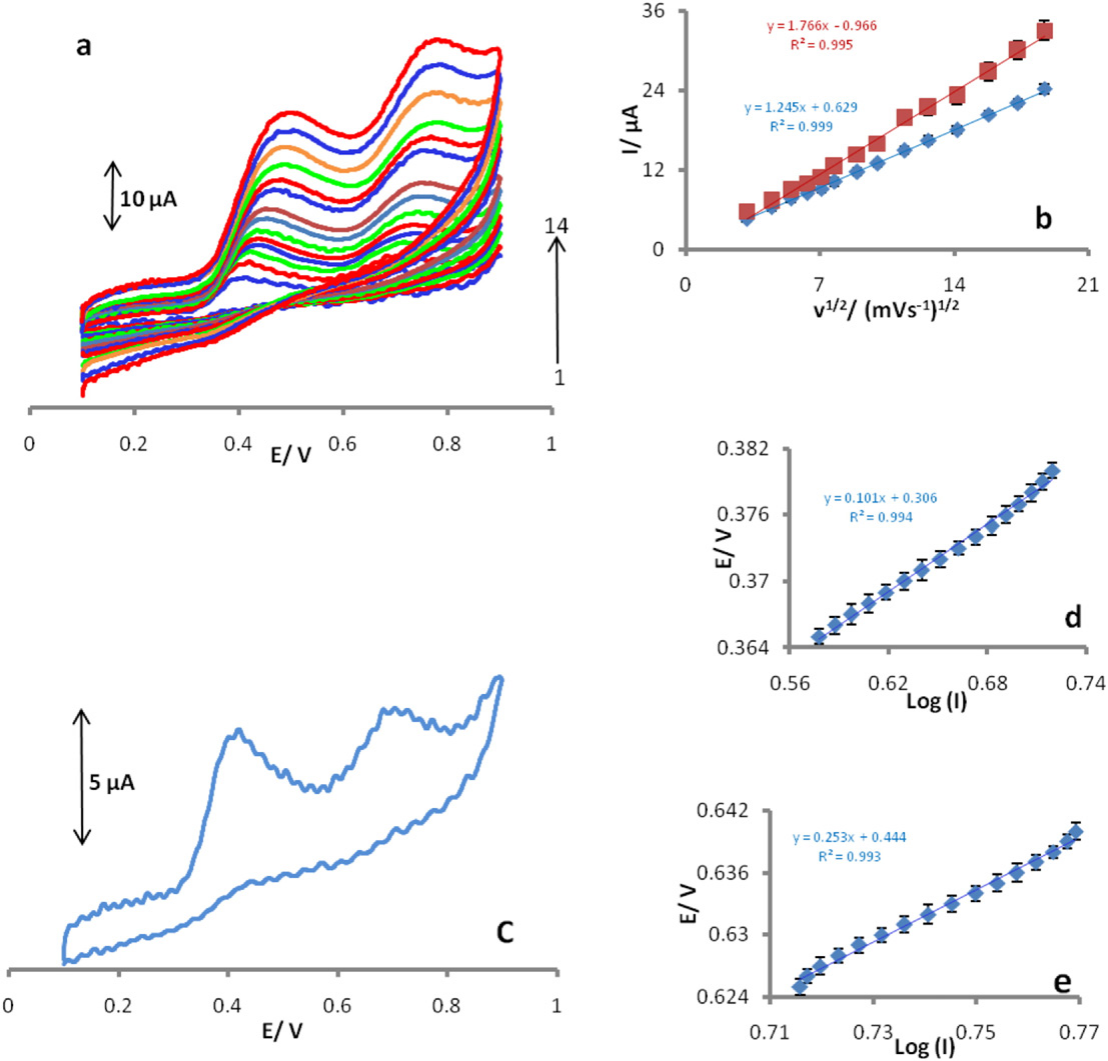
CVs of 0.25 mM of UA and 0.45 mM of FA with different scan rates (from 1 to 14) 10, 20, 30, 40, 50, 60, 80, 100, 130, 160, 200, 250, 300, and 350 mV s^− 1^at POMANS-MWCNT/GPE (a), Plot of peak currents vs. square root of scan rate for UA and FA (b). Cyclic voltammogram (at 20 mV s^− 1^) of a POMANS-MWCNT/GPE in 0.1 M phosphate buffer (pH 6.0) containing 0.25 mM UA and 0.45 mM FA (c), (d) and (e)show the Tafel plots derived from the Cyclic voltammogram for UA and FA.

The effect of scan rate on the electrocatalytic oxidation of 0.25 mM of UA and 0.45 mM of FA at the POMANS-MWCNT/GPE was investigated by cyclic voltammetry (Fig. 4a).The anodic peak potentials shift towards a more positive potential with increasing scan rates, confirming the kinetic limitation of the electrochemical reaction. The anodic peak currents (I_pa_) for UA and FA increased linearly with the square root (ν^1/2^) of the scan rate of potentials (Fig. 4b), suggesting that a sufficient over potential the reaction is diffusion limited. A Tafel plot is also a useful device for evaluating kinetic parameters [30]. Fig. 4c shows a cyclic voltammogram recorded at 20 mV s^− 1^. The rising part of the voltammogram which is known as the Tafel region and is affected by electron transfer kinetics between UA and FA with POMANS-MWCNTs/GPE. Fig. 4d& e show the Tafel plot which are derived from the points in the Tafel region of the cyclic voltammogram of Fig. 4c. A Tafel slopes of 0.101 V decade^− 1^ and 0.253 V decade^− 1^, obtained in this case, agrees well with the involvement of one electron in the rate determining step of the electrode process, assuming a charge transfer coefficients (α) of 0.42 and 0.77 for UA and FA, respectively [30]. The number of electrons in the overall reaction can also be obtained from the slope of the I_p_ vs. ν^1/2^ plot (Fig. 4b). Using the slopes of these plots and according to the following equation for a totally irreversible diffusion controlled process [31]:

(1) $$I_{p}=3.01\times 10^{5}n{\left[\left(1-\alpha \right)n_{\alpha }\right]}^{1/2}{\mathrm{AC}}_{b}D^{1/2}\;\nu^{1/2}$$

According to Eq. (1) and slopes of Fig. 4b the total number of electrons involved in the anodic oxidations for UA and FA were 1.93 and 2.05, respectively.

### 3.5 Chronocoulometric studies

**Fig. 5:**
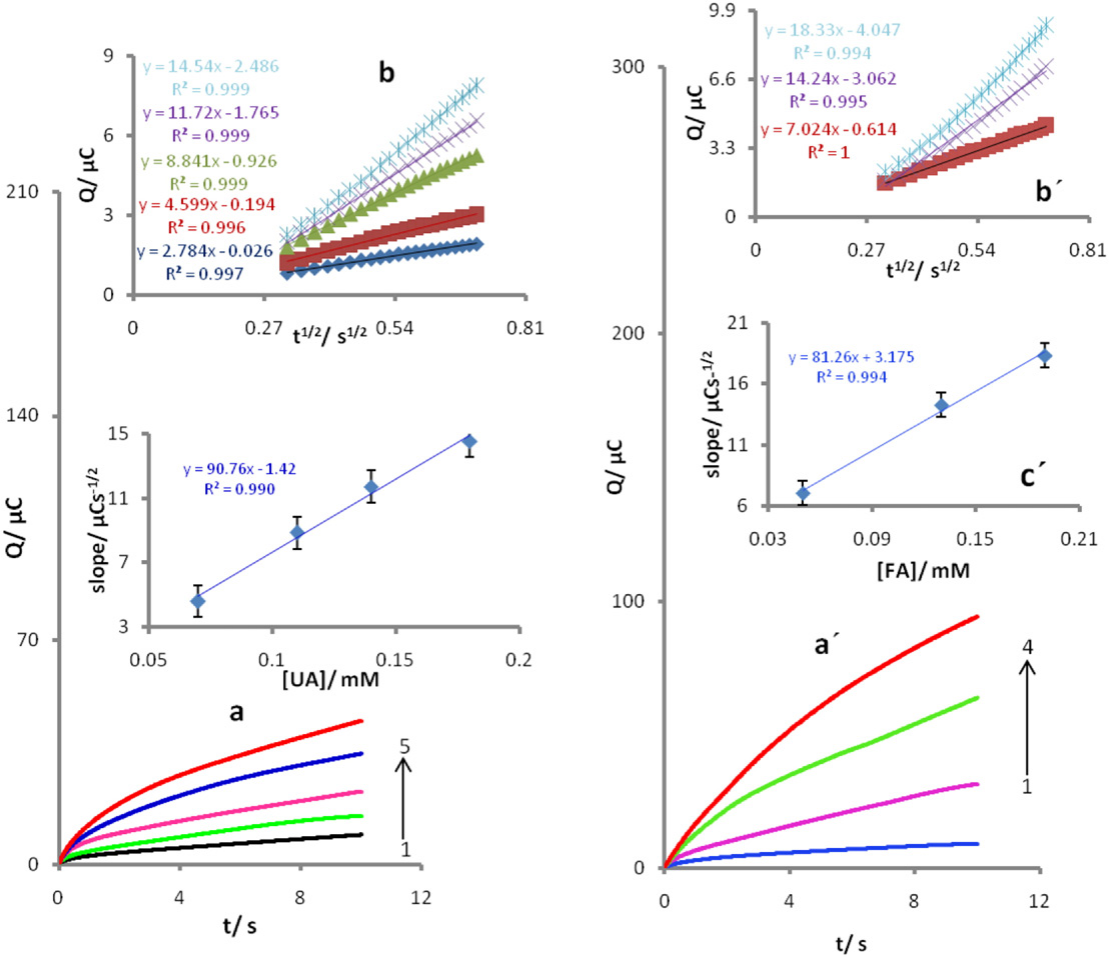
(a) Chronocoulograms obtained at POMANS-MWCNT/GPE in 0.1 M PBS (pH 6.0) for different concentrations of UA. The numbers 1–5 correspond to 0, 0.07, 0.11, 0.14, and 0.18 of UA. (b) plots of Q vs. t^1/2^obtained from chronoamperograms 2–5. (c) Plot of the slope of the straight lines against UA concentration. Similarly, chronocoulometric response of the modified POMA/MWCNT/GPE in 0.1Mphosphate buffer solution (pH 6.0) at potential step of 0.75Vfor different concentrations of FA (a′).The letters 1–4 correspond to 0, 0.05, 0.13 and 0.19 mM FA. (b′) plots of Q versus t^1/2^ obtained from the chronocoulograms and(c′) plot of the straight lines against the FA concentration.

The chronocoulometry as well as the other electrochemical methods was also employed for the investigation of electrode processes at chemically modified electrodes. Chronocoloumetric studies of UA at POMANS-MWCNT/GPE were done by setting the working electrode potential at 350 mV for various concentrations of UA (Fig. 5a). For an electroactive material of UA with a diffusion coefficient of D, the current for the electrochemical reaction, with a mass transport limited rate, is described by the Cottrell equation [30]. Under diffusion control, a plot of Q versus t^1/2^ will be linear (Fig. 5b), and from the slope versus concentration (Fig. 5c) the value of D can be obtained. The value of the D for UA was found to be 2.76 × 10^− 5^ cm^2^ s^− 1^.Similarly, by setting the working electrode potential at 750 mV for FA and carried out the Steps, value of the D for FA was found to be 3.45 × 10^− 5^ cm^2^ s^− 1^(Fig. 5a′, b′ and c′).

### 3.6 Calibration curve and limit of detection

**Fig. 6:**
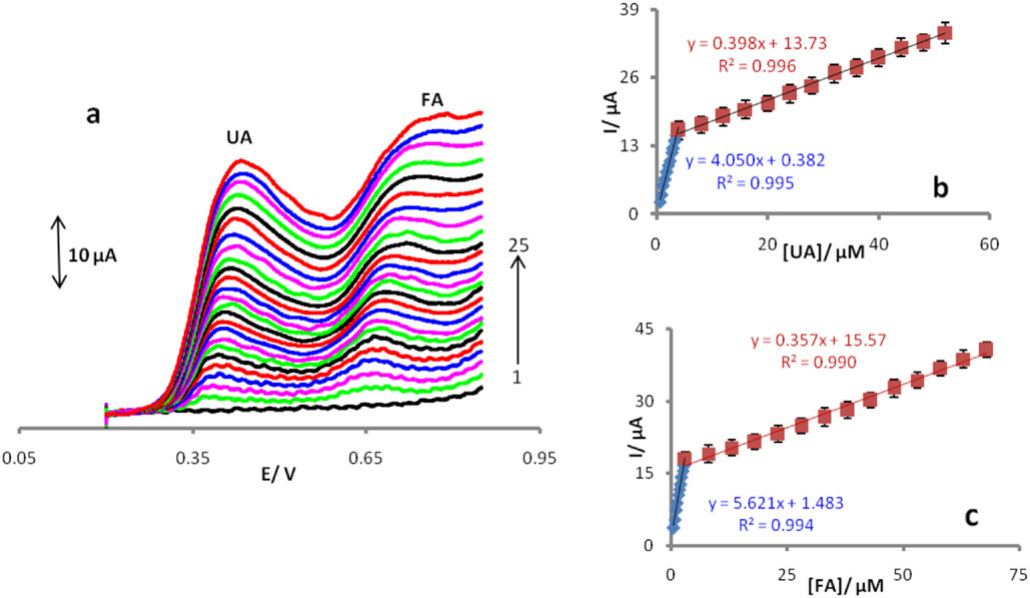
(a) Linear sweep voltammograms of POMANS-MWCNT/GPE 0.1 mol/L PBS (pH 6.0) containing different concentrations of UA and FA. The numbers 1–25 correspond to: 0, 0.6, 0.9, 1.2, 1.5, 1.8, 2.1, 2.4, 2.7, 3, 3.3, 3.6, 4, 8, 12, 16, 20, 24, 28, 32, 36, 40, 44, 48 and 52 μM of UA and 0, 0.5, 0.75, 1, 1.25, 1.5, 1.75, 2, 2.25, 2.5, 2.75, 3, 8, 13, 18, 23, 28, 33, 38, 43, 48, 53, 58, 63 and 68 μM of FA. (b) Represents the variations of anodic peak currents vs. uric acid concentration. (c) Represents the variations of anodic peak currents vs. folic acid concentration.

The results of the voltammetric studies for various concentrations of UA and FA on the surface of POMANS-MWCNTs/GPE, which were obtained under the above mentioned optimum experimental conditions, are shown in Fig. 6a.The oxidation currents of UA showed linear dependences with its concentration over two intervals in the ranges of 0.6 to 4.0 μM (Eq. (2)) and 4.0 to 52.0 μM (Eq. (3)):

(2) $${\mathrm{\Delta I}}_{\mathrm{pa}}\;\left(\mathrm{\mu A}\right)=4.050\;C_{\mathrm{UA}}\left(\mathrm{\mu M}\right)+0.382\;\left(R^{2}=0.995\right)$$

(3) $${\mathrm{\Delta I}}_{\mathrm{pa}}\;\left(\mathrm{\mu A}\right)=0.398\;C_{\mathrm{UA}}\left(\mathrm{\mu M}\right)+13.730\;\left(R^{2}=0.996\right)$$

The results for FA also showed two linear intervals in the ranges of 0.5 to 3.0 μM (Eq. (4)) and 3.0 to 68.0 μM (Eq. (5)):

(4) $${\mathrm{\Delta I}}_{\mathrm{pa}}\;\left(\mathrm{\mu A}\right)=5.621\;C_{\mathrm{FA}}\left(\mathrm{\mu M}\right)+1.483\;\left(R^{2}=0.994\right)$$

(5) $${\mathrm{\Delta I}}_{\mathrm{pa}}\;\left(\mathrm{\mu A}\right)=0.357\;C_{\mathrm{FA}}\left(\mathrm{\mu M}\right)+15.570\;\left(R^{2}=0.990\right)$$

**Fig. 7:**
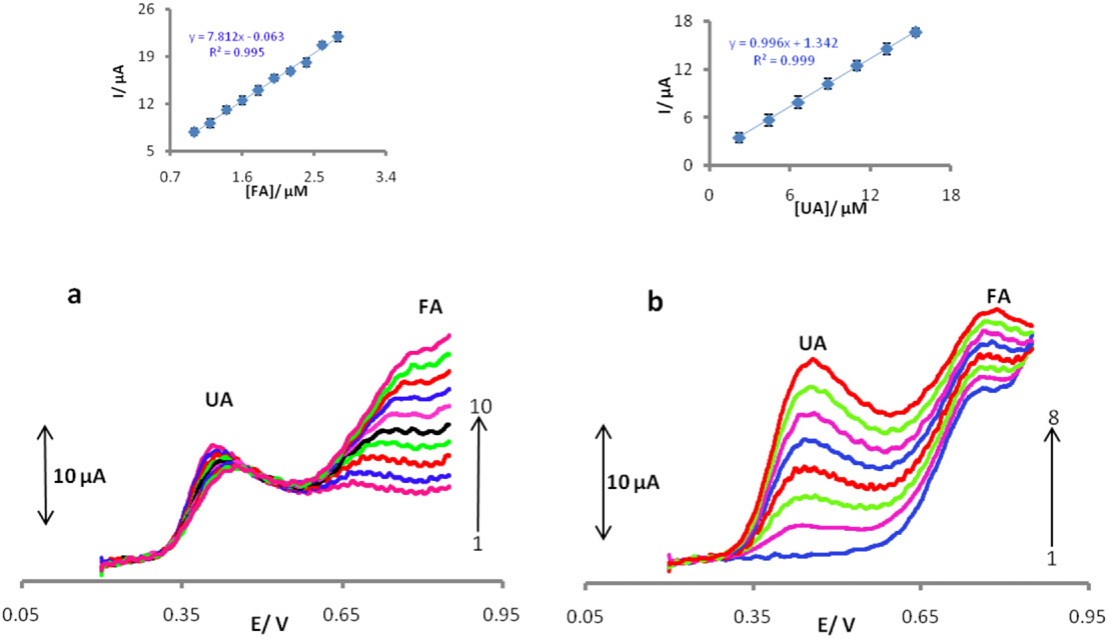
LSVs for the mixture containing UA and FA in 0.1 M (pH 6.0) at POMANS-MWCNT/GPE modified electrode. (a) UA (3 μM) and FA (1–10: 1, 1.2, 1.4, 1.6, 1.8, 2, 2.2, 2.4, 2.6, 2.8 μM). (b) FA (2 μM) and UA (1–8: 0, 2.2, 4.4, 6.6, 8.8, 11, 13.2, 15.4 μM).

The decreases of sensitivity (slope) in the second linear ranges are likely to be due to kinetic limitation. Detection limits (S/N = 3) are obtained as 0.157 μM (UA) and 0.113 μM (FA).The other performance this sensor, ability for simultaneous voltammetric detection of the trace amounts of UA and FA. Fig. 7a displays LSVs recording in various concentrations of FA (from 1μMto 2.8 μM) in the presence of a constant concentration of 3 μM of UA. Fig. 7b shows LSVs of various UA concentrations (same as Fig. 7a for FA) in the presence of 2 μM of FA on the surface of POMANS-MWCNT/GPE.

### 3.7 Analytical applications

In order to evaluate the applicability of the proposed method, the modified electrode was used for simultaneous determination of the content of UA and FA in urine and serum by standard addition method. In order to avoid the interferences of the real samples, from real sample matrices was used from standard addition method; however, human serum and urine samples were centrifuged before the experiment. The 1.0 mL urine or serum samples were diluted 50 times with PBS before the measurements to prevent the matrix effect of real samples. The results were presented in Table 1. To ascertain the correctness of the results, the diluted samples mentioned above were spiked with certain amounts of UA and FA and then were detected. The results of recovery evaluations showed an average recovery of 100.45% for UA and 99.6% for FA added to the human urine, also 101.96% for UA and 102.08% for FA added to the human serum.

**Table 1:** The application of POMANS-MWCNT/GPE for simultaneous determination of UA and FA in real samples by standard addition method (*n* = 5).

Sample	Spiked (μM)		Found (μM)		Recovery (μM)		RSD (%)	
	UA	FA	UA	FA	UA	FA	UA	FA
Human urine	0	0	10.3	ND	–	–	2.7	–
	5.0	3.0	15.1	2.9	98.7	96.7	2.1	2.4
	10.0	5.0	20.7	4.8	102.0	96.0	1.9	2.7
	15.0	10.0	24.9	10.3	98.4	103.0	1.9	2.3
	20.0	15.0	30.8	15.4	101.7	102.7	1.6	2.5
Human blood serum	0	0	5.4	ND	–	–	3.6	–
	5.0	3.0	10.2	3.1	98.1	103.3	1.7	2.1
	10.0	5.0	15.7	5.2	101.9	104.0	1.4	2.5
	15.0	10.0	21.3	10.3	104.4	103.0	1.8	2.9
	20.0	15.0	26.3	14.7	103.5	98.0	2.6	2.8

RSD relative standard deviation, ND not detected.

## 4 Conclusions

Poly ortho-methoxyaniline nanostructures were synthesized by a new method of two-phase and were used for fabrication of an electrochemical sensor for simultaneous determination of uric acid and folic acid. The obtained results revealed a considerable enhancement in the electrochemical performance of the modified electrode towards POMANS. The prepared electrode with desirable electrochemical features such as good reproducibility and repeatability, high sensitivity, wide linear dynamic range, low detection limit, acceptable longtime stability of the electrode response and low cost together with the easy preparation of the modified electrode. Finally, the nanostructure probe in modified electrode showed satisfactory results when applied for the determination of these substances in human urine and human blood serum as real samples.

## References

[1] Ates M. (2013). A review study of (bio)sensor systems based on conducting polymers. Mater. Sci. Eng. C.

[2] Li X., Wang Y., Yang X., Chen J., Fu H., Yo T.C. (2012). Conducting polymers in environmental analysis. Trends Anal. Chem..

[3] Macinnes D., Font L. (1988). Poly-o- methoxyaniline: a new soluble conducting polymers. Synth. Met..

[4] Karimi-Maleh H., Tahernejad-Javazmi F., Atar N., Lufi-Yola M., Gupta V.K., Ensafi A.A. (2015). A novel DNA biosensor based on a pencil graphite electrode modified with polypyrrole/functionalized multi walled carbon nanotubes for determination of 6-mercaptopurine anticancer drug. Ind. Eng. Chem. Res..

[5] Ertan B., Eren T., Ermis I., Saral H., Atar N., Yola M.L. (2016). Sensitive analysis of simazine based on platinum nanoparticles on polyoxometalate/multi-walled carbon nanotubes. J. Colloid Interface Sci..

[6] Komsiyska L. (2005). Ts. Tsacheva, V. Tsakova, electrochemical formation and copper modification of poly-*o*-methoxyaniline. Thin Solid Films.

[7] Fischer A.E., McEvoy T.M., Long J.W. (2009). Characterization of ultrathin electroactive films synthesize d via the self-limiting electropolymerization of *o*-methoxyaniline. Electrochim. Acta.

[8] Mazur M., Michota-Kaminska A., Bukowska J. (2007). Surface-catalyzed growth of poly(2-methoxyaniline) on gold. Electrochim. Acta.

[9] Aitout R., Belgaid A., Makhloufi L., Saidani B. (2006). Synthesis of conducting poly-ortho-methoxy-aniline films onto an inert support (Plexiglas) and their modification with gold by cementation: electrocatalytic tests versus oxidation of hydrazine and proton reduction. React. Funct. Polym..

[10] Raoof J.B., Hosseini S.R., Rezaee S. (2014). Preparation of Pt/poly(2-methoxyaniline)/multi-walled carbon nanotube nanocomposite and its application for electrocatalytic oxidation of methanol. J. Mol. Liq..

[11] Komsiyska L., Tsakova V. (2006). Ascorbic acid oxidation at non modified and copper-modified polyaniline and poly- ortho-methoxyaniline coated electrodes. Electroanalysis.

[12] Abdelwahab A.A., Shim Y.B. (2015). Simultaneous determination of ascorbic acid, dopamine, uric acid and folic acid based on activated graphene/MWCNT nanocomposite loaded Au nanoclusters. Sensors Actuators B.

[13] Noroozifar M., Khorasani-Motlagh M., Zareian-Jahromi F., Rostami S. (2013). Sensitive and selective determination of uric acid in real samples by modified glassy carbon electrode with holmium fluoride nanoparticles/multi-walled carbon nanotube as a new biosensor. Sensors Actuators B Chem..

[14] Noroozifar M., Khorasani-Motlagh M., Taheri A. (2010). Preparation of silver hexa cyanoferrate nanoparticles and its application for the simultaneous determination of ascorbic acid, dopamine and uric acid. Talanta.

[15] Mazloum-Ardakani M., Sabaghian F., Khoshroo A., Naeimi H. (2014). Simultaneous determination of the concentrations of isoproterenol, uric acid, and folic acid in solution using a novel nanostructure- based electrochemical sensor. Chin. J. Catal..

[16] Noroozifar M., Khorasani-Motlagh M., Akbari R., Bemanadi-Parizi M. (2011). Simultaneous and sensitive determination of a quaternary mixture of AA, DA, UA and Trp using a modified GCE by iron ion-doped natrolite zeolite-multiwall carbon nanotube. Biosens. Bioelectron..

[17] Xiao F., Ruan C., Liu L., Yan R., Zhao F., Zeng B. (2008). Single-walled carbon nanotube-ionic liquid paste electrode for the sensitive voltammetric determination of folic acid. Sensors Actuators B Chem..

[18] Jamali T., Karimi-Maleh H., Khalilzadeh M.A. (2014). A novel nanosensor based on Pt:Co nano alloy ionic liquid carbon paste electrode for voltammetric determination of vitamin B9 in food samples. LWT- Food Sci. Technol..

[19] Nie T., Lu L., Bai L., Xu J., Zhang K., Zhang O., Wen Y., Wu L. (2013). Simultaneous determination of folic acid and uric acid under coexistence of l-ascorbic acid using a modified electrode based on poly(3,4-ethylenedioxythiophene) and functionalized single-walled carbon nanotubes composite. Inter. J. Electrochem. Sci..

[20] Akbari R., Noroozifar M., Khorasani-Motlagh M., Taheri A. (2010). Simultaneous determination of ascorbic acid and uric acid by a new modified carbon nanotube-paste electrode using chloromercuriferrocene. Anal. Sci..

[21] Noroozifar M., Khorasani-Motlagh M., Khaleghian-Moghadam R., Ekrami-Kakhki M.S., Shahraki M. (2013). Incorporation effect of nanosized perovskite LaFe_0.7_Co_0.3_O_3_on the electrochemical activity of Pt nanoparticles-multi walled carbon nanotube composite toward methanol oxidation. J. Solid State Chem..

[22] Noroozifar M., Khorasani-Motlagh M., Hassani-Nadiki H., Hadavi M.S., Foroughi M.M. (2014). Modified fluorine-doped tin oxide electrode with inorganic ruthenium red dye-multiwalled carbon nanotubes for simultaneous determination of a dopamine, uric acid, and tryptophan. Sensors Actuators B Chem..

[23] Yola M.L., Atar N. (2014). A novel voltammetric sensor based on gold nanoparticles involved in *p*-aminothiophenol functionalized multi-walled carbon nanotubes: application to the simultaneous determination of quercetin and rutin. Electrochem. Acta.

[24] Golestanifar F., Karimi-Maleh H., Atar N., Aydogdu A., Ertan B., Taghavi M., Yola M.L., Ghaemy M. (2015). Voltammetric determination of hydroxylamine using a ferrocene derivative and NiO/CNTs nanocomposite modified carbon paste electrode. Int. J. Electrochem. Sci..

[25] Noroozifar M., Khorasani-Motlagh M., Taheri A. (2011). Determination of cyanide in wastewaters using modified glassy carbon electrode with immobilized silver hexacyanoferrate nanoparticles on multiwall carbon nanotube. J. Hazard. Mater..

[26] Noroozifar M., Khorasani-Motlagh M., Tavakkoli H. (2011). Preparation of tetra heptylammonium iodide-iodine graphite-multiwall carbon nanotube paste electrode: electrocatalytic determination of ascorbic acid in pharmaceuticals and foods. Anal. Sci..

[27] Noroozifar M., Khorasani-Motlagh M., Tavakkoli H. (2012). Determination of ascorbic acid by a modified multiwall carbon nanotube paste electrode using cetrimonium iodide/iodine. Turk. J. Chem..

[28] Yola M.L., Eren T., Ater N. (2014). Biosens. Bioelectron.

[29] Savale P.A., Shirale D.J., Datta K., Ghosh P., Shirsat M.D. (2007). Synthesis and characterization of poly (o-anisidine) films under galvanostatic conditions by using ECP technique. Int. J. Electrochem. Sci..

[30] Bard A.J., Faulkner L.R. (2001).

[31] Antoniadou S., Jannakoudakis A.D., Theodoridou E. (1989). Electrocatalytic reaction on carbon fiber electrodes modified by hemine II electro-oxidation of hydrazine. Synth. Met..

